# Military Personals

**Published:** 1918-09

**Authors:** 


### MILITARY PERSONALS

Major Marshal Clinton, Buffalo surgeon, who has been attached to base hospital No. 23, near the French front, has just been promoted to be lieutenant. It is understood the Buffalo officer has been detached from base hospital service and is now on duty just behind the first line trenches.

Dr. Marshall L. Hillsman of Little Valley, who enlisted in the medical department, has received a commission as first lieutenant and will leave for Oglethorpe, Ga., on August 13th.

To Camp Crane, Lieut. Roy Marshall, Rome.

To Camp Gordon, Capt. Herbert Sperry, Rochester; Lieut. Arthur Glover, Buffalo.

To Camp Grant, Lieut. Wm. McCanty, Buffalo.

To Camp Jackson, Lieut. James Short, Buffalo.

To Camp Logan, Lieut. Abraham Lebendig, Rochester.

To Camp McArthur, Lieut. Floyd Breese, Elmira.

To Camp Sevier, Capt. Wm. Reid, Rome.

To Fort Des Moines, Lieut. Harry Wronker, Rochester.

To Fort Oglethorpe, Capt. Francis O'Brien, Auburn; Lieuts. Raymond Laport, Buffalo; Halley Hammond, Franklinville; Clarence Fessenden, Fulton; Frankling Knope, Rochester.

To Gas Defense Plant, Long Island City, Capt. A. L. Benedict, Buffalo.

To Mineola, Capts. Edwin Ingersoll, Rochester; Samuel Munford, Ithaca.

To New York City, Lieuts. Elmer Clarke, Buffalo; Chas. Steinhauser, Rochester.

To Camp Jackson, Lieut. Edward Frost, Buffalo.

To Camp Wheeler, Lieut. Carl Zimmerman, Elmira.

To New Haven, Capt. Frederick Bowen, Mt. Morris.

To Williamsbridge, Capt. Norris Orchard, Rochester.

To Camp Shelby, Lieut. Henry Spofford, Batavia.

To Camp Zachery Taylor, Lieut. Chas. Maggie, Rochester.

To Fort Oglethorpe, Ga., Lieuts. Harry Scott, Batavia; Lyman Lewis, Belmont; Howard Hoehman, Vincent Moscatee, Buffalo; Wm. Jones, Jamestown; Kenneth Smith, Edward Twist, Lackawanna; Edward Amsler, Norman Pfaff, Rochester; Jacob Dewees, Buffalo; Geo. Peddle, Perry.

To Rochester Institute, Lieut. Joseph Laduca, Buffalo; Capt. Francis Goldsborough, Buffalo; Lieuts. Geo. Davis, Arcade; Harold Patterson, Buffalo; Louis Preston, Canisteo; Capt. Wm. Thomson, Warsaw and later to Base Hospital, Camp Sheridan, Ala.

To Camp Reed General Hospital, Capt. Herman Raymond, Collins; Lieut. Frank Walz, Buffalo.

To Camp Devens, Lieut. Melen, Rochester.

To Camp Dix, Lieut. Francis Kujawa, Buffalo.

To Camp Hancock, Capt. Gordon Priestman, Willard.

To Camp Lee, Lieut. Clayton Axtell, Deposit.

To Camp Upton, Lieut. Jeremiah Morin, Rochester.

To Columbus Barracks, Lieut. Rollin Hadley, Collins.

To Fort Des Moines, Major Martin Tinker, Ithaca.

To Richmond, Va., Capt. Gordon Graham, Rochester.

Major G. M. Davis, who practiced in Welland and volunteered for service in the Canadian Army at the beginning of the war, is believed to have been drowned when the hospital ship Landovery Castle, was torpedoed.

